# Evaluation of Petrifilms^TM^ as a diagnostic test to detect bovine mastitis organisms in Kenya

**DOI:** 10.1007/s11250-012-0286-y

**Published:** 2012-10-30

**Authors:** George K. Gitau, Royford M. Bundi, John Vanleeuwen, Charles M. Mulei

**Affiliations:** 1 Department of Clinical Studies, Faculty of Veterinary Medicine, University of Nairobi, P.O. Box 29053-00625, Nairobi, Kenya; 2 Centre for Veterinary Epidemiologic Research, Department of Health Management, Atlantic Veterinary College, University of Prince Edward Island, 550 University Avenue, Charlottetown, PE Canada C1A 4P3

**Keywords:** Dairy cattle, Mastitis, Laboratory culture, Petrifilms^TM^, Test evaluation, Kenya

## Abstract

The study purpose was to validate Petrifilms^TM^ (3M Microbiology, 2005) against standard culture methods in the diagnosis of bovine mastitis organisms in Kenya. On 128 smallholder dairy cattle farms in Kenya, between June 21, 2010 and August 31, 2010, milk samples from 269 cows that were positive on California Mastitis Test (CMT) were cultured using standard laboratory culture methods and Petrifilms^TM^ (Aerobic Count and Coliform Count –3M Microbiology, 2005), and results were compared. *Staphylococcus aureus* was the most common bacterium isolated (73 % of samples). Clinical mastitis was found in only three cows, and there were only two Gram-negative isolates, making it impossible to examine the agreement between the two tests for Gram-negative- or clinical mastitis samples. The observed agreement between the standard culture and Petrifilm^TM^ (3M Microbiology, 2005) results for Gram-positive isolates was 85 %, and there was fair agreement beyond that expected due to chance alone, with a kappa (*κ*) of 0.38. Using culture results as a gold standard, the Petrifilms^TM^ had a sensitivity of 90 % for Gram-positive samples and specificity of 51 %. With 87 % of CMT-positive samples resulting in Gram-positive pathogens cultured, there was a positive predictive value of 93 % and a negative predictive value of 43 %. Petrifilms^TM^ should be considered for culture of mastitis organisms in developing countries, especially when Gram-positive bacteria are expected.

## Introduction

Standard culture methods have been widely used by laboratories for determining the bacteria causing mastitis for control programs (Villanueva et al. 1991). However, small laboratories in remote parts of developing countries are often not able to maintain the quality control protocols necessary for obtaining reliable results from these tests (Villanueva et al. 1991). Therefore, there is a need for a quick, inexpensive, easy-to-perform diagnostic test that could reliably identify the major pathogen categories that cause mastitis, thus enabling a farmer to choose an appropriate antibiotic treatment regimen especially among the smallholder dairy farmers in highlands of tropical Africa.

Recently, 3M (3M Microbiology, 2005) developed a ready-made culture Petrifilm^TM^ for quick field diagnosis of bacterial infections, including mastitis in cattle. The Petrifilm^TM^ Aerobic Count (AC) and Petrifilm^TM^ Coliform Count (CC), when used together, have the capability of distinguishing mastitis samples with no bacterial growth, Gram-negative infections and Gram-positive infections which can benefit from treatment. Our study objective was to validate Petrifilms^TM^ in the diagnosis of bovine mastitis organisms in smallholder dairy cattle in Kenya, as compared to the standard culture methods, which may lead to confident use of Petrifilms^TM^ in low-income countries.

## Materials and methods

The study was carried out between June 21, 2010 and August 31, 2010 on 64 zero-grazing farms in Mukurweini District of Nyeri County and 64 partially zero-grazing farms in greater Nakuru District of Nakuru County in Kenya, where dairy farming is an important enterprise (Ministry of Livestock Development 2008). In Nakuru, a simple random selection was employed at the farm level, using a sampling frame of the district dairy farms provided by the District Livestock Production Officer. In Mukurweini, a convenient sampling procedure was used to select farms, to reduce costs for sampling, as researchers were on the farms for other reasons (Dohoo et al. 2012). The study farms selected were visited twice during the study period to increase the sample size, as researchers were on the farms for other reasons.

Within the herds, cows were eligible for the study if they were lactating. Any farm with less than five lactating cows had all the cows selected for the study. In the farms that had more than five lactating cows, the lactating cows were systematically randomly selected by examining alternate cows in the crush as follows one, three, five … in that order. At all times, less than ten cows were examined in every farm.

From quarters that were positive on the California Mastitis Test (score of 1+ or more on a scale of negative, trace, 1+, 2+, or 3+), milk samples were collected, after routine teat cleaning and disinfection using 70 % alcohol, as described by the National Mastitis Council (1999). If more than one quarter was California Mastitis Test (CMT)-positive in the same cow, a composite milk sample for culture was taken from all CMT-positive quarters. The composite sample taken from the different quarters comprised one milk stream from each quarter which was considered to be nearly the same volume. The first stream of milk from each quarter was discarded prior to sampling for both CMT and cultures. The milk samples were refrigerated for a maximum of 96 h (4 days) until they were transported on ice packs in a cool box to the Clinical Microbiology Laboratory, Department of Clinical Studies, Faculty of Veterinary Medicine, University of Nairobi.

At the laboratory, bacteriological cultures were performed on the milk samples according to the Laboratory Handbook on Bovine Mastitis (National Mastitis Council 1999). A 10-μL aliquot of each milk sample was streaked onto the surface of 5 % sheep blood agar and MacConkey agar plates. The plates were incubated at 37 °C for 18–24 h. Where growth occurred, the cultures were examined microscopically for Gram reaction and classified as either Gram-positive or Gram-negative, and biochemical tests, such as catalase and citrate tests, were conducted to determine the genus and species of isolates. Samples that had two colony types were considered as mixed growth and samples with three or more colony types were considered contaminated.

Petrifilm^TM^ (3M Microbiology, 2005) cultures were also conducted, using the following process. The milk samples were first diluted with sterile water at the ratio of 1:10 (milk/water). Dilution was done to produce better readability of the Petrifilms, as suggested by McCarron et al. (2009). After mixing the diluted milk sample, a 1-mL aliquot of each diluted milk sample was placed on the Petrifilm^TM^ AC (aerobic count) and CC (coliform count) plates. All plates were incubated at 37 °C for 24 h. The colonies on each pair of Petrifilms^TM^ were counted. As suggested by McCarron et al. (2009), the sample was categorized as positive, if there were 20 or more colonies present on the CC plates or five or more colonies on the AC plates. Positive colony growth on both the AC and CC Petrifilms^TM^ was classified as Gram-negative because coliforms will also grow on the AC plate but only coliforms will grow on the CC plate (although mixed growth could lead to growth on both—see “Discussion”). Colony growth on only the AC Petrifilm^TM^ was classified as a Gram-positive organism. If there were <20 colonies on CC Petrifilms^TM^ and <5 colonies on the AC plates, the sample was categorized as no growth. All of the readings for the Petrifilms^TM^ and standard culture tests were done by the same blinded technician.

The data were entered into an Excel spreadsheet and exported to the statistical package, Genstat for statistical analyses. Kappa (*κ*) was used to test the level of agreement between the Petrifilm^TM^ and culture results, beyond that expected due to chance alone. Using culture results as the gold standard, sensitivity, specificity, positive predictive value (PPV), and negative predictive value (NPV) of the Petrifilms^TM^ were calculated, including 95 % confidence intervals (95 % CI) (Dohoo et al. 2009).

## Results

Of the 269 cows involved in the study, 48 % (95 % CI, 39 to 57 %) and 52 % (95 % CI, 44 to 60 %) were positive on CMT and sampled for milk cultures for the first (*n* = 130) and second (*n* = 139) samplings. *Staphylococcus aureus* was the most common bacterium isolated (Table 1). Clinical mastitis was found in only three cows.

**Table 1:** Laboratory culture results of CMT-positive dairy cow udders for the first (*n* = 130) and second (*n* = 139) visits from Mukurweini and greater Nakuru districts, Kenya—June to August 2010

Organism	First visit frequency (proportion)	Second visit frequency (proportion)
*Staphylococcus aureus*	89 (0.68)	108 (0.77)
*Streptococcus agalactiae*	10 (0.08)	4 (0.03)
Other streptococci	6 (0.05)	7 (0.05)
Coagulase negative staphylococci	4 (0.03)	0 (0)
*Corynebacterium bovis*	3 (0.02)	3 (0.03)
Mixed growth (staphylococci and streptococci)	2 (0.02)	3 (0.03)
*Klebsiella*	1 (0.01)	1 (0.01)
No growth	15 (0.12)	13 (0.08)
Total	130 (1.0)	139 (1.0)

With only one Gram-negative sample in each of the first and second samplings, we were unable to examine the agreement between the two tests for Gram-negative samples, and so subsequent results are for Gram-positive test results only.

Table 2 shows that there was 85 % agreement (95 % CI, 81 to 89 %) between the Petrifilm^TM^ and culture results for all samples combined. There was fair agreement beyond that expected due to chance alone between the two tests, with a kappa of 0.38 (95 % CI, 0.32 to 0.40). Using culture results as the gold standard, sensitivity of the Petrifilm^TM^ test was 90 % (95 % CI, 86 to 94 %) and specificity was 51 % (95 % CI, 34 to 68 %). With 87 % (95 % CI, 83 to 91 %) of CMT-positive samples resulting in Gram-positive pathogens cultured (estimated true prevalence), positive predictive value was 93 % (95 % CI, 90 to 96 %) and negative predictive value was 43 % (95 % CI, 28 to 58 %).

**Table 2:** Comparison of Gram-positive results from culture and Petrifilms^TM^ for CMT-positive milk samples collected from Mukurweini and greater Nakuru districts, Kenya—June to August 2010

	Petrifilm^TM^ positive^a^	Petrifilm^TM^ negative^b^	Total
Culture positive^c^	210	24	234
Culture negative^d^	17	18	35
Total	227	42	269

^a^Petrifilm positive reflects samples that were Gram-positive growth

^b^Petrifilm negative reflects samples that were either no growth, mixed growth, or Gram-negative growth

^c^Culture positive reflects growth on blood agar

^d^Culture negative reflects no growth on blood agar

## Discussion

For the Gram-positive mastitis isolates from smallholder dairy farms in Kenya, there was good agreement between the Petrifilm^TM^ and culture results, with very good sensitivity and PPV. This can be replicated in the smallholder dairy farmers in highlands of tropical Africa. The specificity and NPV were less than ideal, indicating that when the Petrifilm^TM^ determined a CMT-positive milk sample to not be infected, the owners should probably retest the cow to confirm that the cow is truly not infected. However, the prevalence of Gram-positive infections in a population will have an impact on the PPV and NPV values, whereby, if the prevalence was to be lower, the low specificity would likely lead to a poorer PPV.

Our observed agreement (85 %) supports the results of several other studies elsewhere which indicated that Petrifilms^TM^ appear to be about 80 % accurate in differentiating Gram-positive and Gram-negative pathogens (Lago et al. 2006; McCarron et al. 2009; Pol et al. 2009; Rodrigues et al. 2009) and better than the agreement obtained (63 %) by Ruegg et al. (2009). While the observed agreement was good, the kappa was still only fair, mostly because the Petrifilm^TM^ called some culture-positive samples negative (undetected organisms). Perhaps, a lower cutoff for classifying a sample as positive on Petrifilm^TM^ would have lead to a better kappa. Also, some culture-negative samples were classified as positive on Petrifilms^TM^, perhaps from contamination when diluting and inoculating Petrifilms^TM^.

Our study had very few Gram-negative isolates, likely because the study occurred during the dry season in Kenya. Udders are typically substantially cleaner in the dry season. Data on the agreement and sensitivity/specificity of the Petrifilm^TM^ for Gram-negative samples within the context of smallholder dairy farms would be important to determine before Petrifilms^TM^ were used in small laboratories in remote parts of developing countries. Also, if different Gram-positive pathogens were isolated, different sensitivity and specificity results could possibly be obtained. A larger sample size with analyses by different pathogens would help elucidate this possibility.

Mixed growth of Gram-positive bacteria would not be differentiated on Petrifilms, and similarly mixed growth of Gram-negative bacteria would not be differentiated on Petrifilms. If there was a mixed growth of a Gram-positive bacterium and a Gram-negative bacterium, it would be classified as Gram-negative. However, the culture results showed limited numbers of samples with mixed growth (2 %); therefore, implications of mixed growth samples to our results are minimal.

## Conclusions

Compared to culture results, the Petrifilm^TM^ results for Gram-positive udder infections (73 % *S. aureus*) showed high sensitivity (90 %), positive predictive value (93 %) and agreement (85 %), and fair agreement (kappa = 0.38) beyond that expected due to chance alone. These results provide preliminary data toward the use of Petrifilms^TM^ in low-income countries
